# Synthesis of Tungsten Carbides in a Copper Matrix by Spark Plasma Sintering: Microstructure Formation Mechanisms and Properties of the Consolidated Materials

**DOI:** 10.3390/ma16155385

**Published:** 2023-07-31

**Authors:** Tomila M. Vidyuk, Arina V. Ukhina, Alexander I. Gavrilov, Vladislav S. Shikalov, Alexander G. Anisimov, Oleg I. Lomovsky, Dina V. Dudina

**Affiliations:** 1 Institute of Solid State Chemistry and Mechanochemistry SB RAS, Kutateladze Str. 18, Novosibirsk 630090, Russia; auhina181@gmail.com (A.V.U.); gavr_sand@mail.ru (A.I.G.); lomov@solid.nsc.ru (O.I.L.); dina1807@gmail.com (D.V.D.); 2 Khristianovich Institute of Theoretical and Applied Mechanics SB RAS, Institutskaya Str. 4/1, Novosibirsk 630090, Russia; v.shikalov@gmail.com; 3 Lavrentyev Institute of Hydrodynamics SB RAS, Lavrentyev Ave. 15, Novosibirsk 630090, Russia; anis@hydro.nsc.ru

**Keywords:** tungsten carbide, composite, mechanical milling, spark plasma sintering, inert matrix

## Abstract

In this study, the synthesis of tungsten carbides in a copper matrix by spark plasma sintering (SPS) is conducted and the microstructure formation mechanisms of the composite materials are investigated. The reaction mixtures were prepared by the high-energy mechanical milling (MM) of W, C and Cu powders. The influence of the MM time and SPS temperature on the tungsten carbide synthesis in an inert copper matrix was analyzed. It was demonstrated that the milling duration is a critical factor for creating the direct contacts between the W and C reactants and increasing the reactive transformation degree. A WC–W_2_C–Cu composite was fabricated from the W–C–3Cu powder mixture milled for 10 min and subjected to SPS at a temperature of 980 °C for 5 min. The formation of unconventional microstructures with Cu-rich regions is related to inter-particle melting during SPS. The WC–W_2_C–Cu composite showed a promising combination of mechanical and functional properties: a hardness of 300 HV, an electrical conductivity of 24% of the International Annealed Copper Standard, a residual porosity of less than 5%, a coefficient of friction in pair with a WC-6Co counterpart of 0.46, and a specific wear rate of the material of 0.52 × 10^−5^ mm^3^ N^−1^ m^−1^.

## 1. Introduction

A reactive approach to the formation of metal matrix composites (MMCs) is based on the in situ synthesis of the reinforcing phase [1,2,3]. Spark plasma sintering (SPS) is one of the modern methods of obtaining MMCs from powders by means of the pulsed electric current passage and the application of pressure [4]. During reactive SPS, both the synthesis of the reinforcing phases and consolidation of the powders can be completed within a few minutes in a single technological step [5].

The synthesis of a carbide-reinforced MMC is governed by the possible chemical reactions between the metal matrix, carbon, and the carbide-forming element as well as by their mutual solubility [6]. In the W–C–Cu system, the copper matrix is inert to both tungsten and carbon [7]. Copper does not form carbides, and the solubility of carbon in copper is extremely low. Tungsten is insoluble in copper; no intermetallic exists in the Cu–W system. The inertness of the matrix to carbon and the carbide-forming element prevents the diffusion of the reactants in/through the matrix. So, the copper matrix acts as a strong diffusion barrier, hindering the interaction between tungsten and carbon. Thus, the tungsten carbide synthesis is possible only upon the direct contact of the W and C reactant particles. In the W–C system, the formation of WC and W_2_C carbides occurs by the following chemical reactions:

2W + C → W_2_C (1)

W_2_C + C → 2WC (2)

W + C → WC(3)

The W_2_C phase is metastable at room temperature and atmospheric pressure [8,9]. Under conditions of SPS, the W_2_C phase is easily synthesized when the amount of carbon is not sufficient to form WC [9]. 

The possibility of the synthesis of tungsten carbide phases from elements in a copper matrix during thermal annealing (820–940 °C) was shown by Baikalova and Lomovsky [7]. The interactions between the starting components were facilitated by high-energy mechanical milling (MM). MM is a well-known solid state method for preparing the reaction mixtures, which helps to increase both the contact area between the phases and the concentration of defects of the crystalline structure [10]. During SPS, the fine-grained structure of the milled powders is preserved owing to the short holding times. Thus, a combination of MM and SPS is a way of forming nanostructured materials [11]. 

Copper-based materials reinforced by WC particles have high hardness, high electrical and thermal conductivity, and high wear resistance. Due to their attractive properties, WC–Cu compositions are promising as electrical contact materials [12,13], electrodes for resistance welding and electrical discharge machining [14,15], and as heat sink materials for fusion applications [16,17]. WC–Cu composites have been manufactured by non-reactive methods, such as infiltration of molten copper into a porous tungsten carbide preform [18,19], sintering of the ex-situ synthesized WC mixed with copper [12,16,20], and stir casting [13].

To date5, the processes of the structure formation of MMCs during reactive SPS have not been sufficiently studied. Pulse electrical current passing through the sample during SPS can lead to the different physical processes occurring at the interparticle contacts, such as local overheating and the melting of the material. SPS allows obtaining unique structures, which are impossible to achieve by conventional sintering. 

The goal of the present work is to conduct tungsten carbide synthesis in a copper matrix by the SPS of mechanically milled elemental powders and investigate the structure formation mechanisms and properties of the sintered materials. The novelty of this study is in determining the features of the composite microstructures obtained during electric current passage. Along with the applied aspects of the materials synthesis and novel sets of properties, this work aims to obtain the fundamental knowledge on the structure formation of materials produced by SPS.

## 2. Materials and Methods

Tungsten powder (PWT, 99.68%, particle size of 3.5–5 μm), carbon black powder (PM-15, 95%, particle size of 100–200 nm), and electrolytic copper powder (PMS-1, 99.7%, average particle size of 40 μm) were used as the starting materials for the synthesis. The components W–C–Cu were taken in a molar ratio of elements of 1:1:3. The complete conversion of the reactants leads to the formation of a 51 wt.% WC–Cu (37 vol.% WC–Cu) composite. 

The MM of the W–C–3Cu powder mixture was conducted in a high-energy planetary ball mill AGO-2 (ISSCM SB RAS, Novosibirsk, Russia) equipped with water-cooled stainless steel vials. Milling was conducted in an argon atmosphere for 3 and 10 min without any process control agent. Then, milling for 30 s was conducted using ethanol as a process control agent to reduce agglomeration and particle sticking to the surface of the milling balls. The powders were milled for 10 min and rested for 5 min throughout all milling times to avoid excessive temperatures and oxidation. The acceleration of the milling balls (made of steel) was 400 m s^−2^. The diameter of the balls was 8 mm, the mass of the balls was 200 g, and the mass of the powder was 10 g. 

The SPS of the milled powders was conducted using a Labox 1575 apparatus (SINTER LAND Inc., Nagaoka, Japan) under dynamic forevacuum (residual pressure in the chamber 10 Pa). Heating was conducted by passing a pulsed direct electric current through the sample and graphite assembly. The latter consisted of the die and punches. The protective foil lines the inner wall of the die and was also placed between the sample and the flat ends of the punches. The pulse duration was 3 ms. The sequence of pulses was 12 ON:2 OFF. The uniaxial pressure of 40 MPa remained constant throughout the sintering process. The heating rate was 70 °C min^−1^. A die with an inner diameter of 20 mm was used. The sintering temperatures of the W–C–3Cu mixtures were 900 °C and 980 °C. The reference copper sample was consolidated by SPS at the temperature of 700 °C. The holding time at the maximum temperature was 5 min. The temperature was measured by an optical pyrometer (CHINO, Japan) focused on a hole in the die wall located at its mid-plane. 

The X-ray diffraction (XRD) patterns of the milled powders and sintered samples were recorded by means of a D8 ADVANCE powder diffractometer (Bruker AXS, Karlsruhe, Germany) with Cu Kα radiation. The parameters of the crystalline structure (crystallite size, lattice strain, and lattice parameters) and concentrations of the phases were determined using the Rietveld refinement in TOPAS 4.2 software (Bruker AXS, Karlsruhe, Germany). The concentration of the residual carbon in the sintered compacts was calculated from the concentrations of the carbide phases obtained via the Rietveld refinement procedure.

The particles of the mechanically milled powders and cross-sections of the consolidated samples were mounted into phenolic resin using a hot press Mecapress 3 (Presi, Eybens, France) and polished using an automatic polishing machine Mecatech 334 (Presi, Eybens, France). The morphology and the microstructure of the milled agglomerates were studied by scanning electron microscopy (SEM) using a Tabletop TM-1000 Microscope (Hitachi, Tokyo, Japan). The microstructure of the sintered compacts and the morphology of the worn surfaces (the wear tests are described below) were studied using a scanning electron microscope EVO MA15 (Zeiss, Oberkochen, Germany). The back-scattered electron imaging mode was used. The contrast in the obtained images is caused by the differences in the elemental composition of the regions. The elemental mapping of the selected regions of the polished cross-sections and the worn surfaces was conducted by Energy Dispersive Spectroscopy (EDS) using an X-Max 80 mm^2^ unit (Oxford Instruments, Abingdon, UK) attached to a scanning electron microscope EVO MA 15 (Zeiss, Oberkochen, Germany).

The residual porosity of the sintered compacts was measured by image analysis on the cross-sections using ImageJ software 1.52t (30 January 2020) (NIH, Bethesda, USA) from the images obtained by Optical Microscopy using an Axio Scope.A1 Microscope (Zeiss, Oberkochen, Germany).

The electrical conductivity of the sintered compacts was measured by a contactless method using a custom-made set up [21] and is reported as% of the International Annealed Copper Standard (IACS). The Vickers hardness of the sintered samples was measured on the polished cross-sections using a DuraScan 50 hardness tester (EMCO-TEST, Kuchl, Austria) at a load of 1 kg. The average hardness values for each sample were determined from 10 measurements.

The dry sliding wear tests of the sintered compacts were conducted using a UMT-2 machine (Bruker, Karlsruhe, Germany) in ball-on-flat reciprocating motion in two modes, the parameters of which are shown in Table 1. AISI 52100 steel and WC-6Co balls 6.35 mm in diameter were used as the counter-bodies. During testing, the counter-body was kept stationary, while the working specimen maintained the reciprocating sliding movement. Prior to the wear tests, the consolidated samples were mounted into phenolic resin, grinded, and polished. After the wear tests, the volume loss of compacts was determined using Vision64 software (Bruker Nano, Karlsruhe, Germany) on the three-dimensional profiles obtained with a ContourGT-K1 interference profilometer (Bruker Nano, Karlsruhe, Germany). For each mode, 3 tests were made, and the average values of friction coefficient (COF) and volume loss were determined. The average value of specific wear rate was calculated as the ratio of the volume loss value to the product of the applied normal load and the total sliding distance values. 

## 3. Results and Discussion

**Figure 1:**
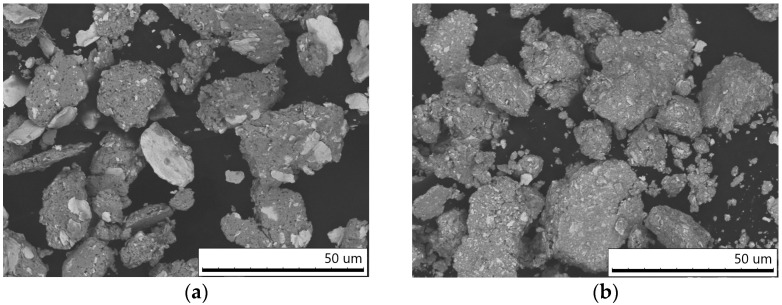

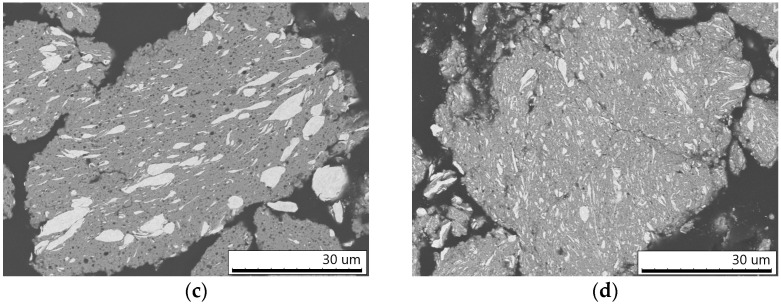
Morphology (**a**,**b**) and microstructure (**c**,**d**) of the W–C–3Cu powder agglomerates mechanically milled for 3 min (**a**,**c**) and 10 min (**b**,**d**). Images were obtained by SEM.

Figure 1a,b shows the morphology of the composite agglomerates of the W–C–3Cu powder mixtures prepared by MM. The starting components were distributed in the volume of agglomerates, which is seen on the cross-sections of the particles (Figure 1c,d). The formation of composite agglomerates is a typical process for the high-energy milling of metal-containing powder mixtures [10]. The W-rich areas are seen as light gray in the micrographs as tungsten is a heavier chemical element than copper. In the mixture milled for 3 min, large tungsten flake-shaped particles were observed, indicating a low mixing level (Figure 1a). In order to mix the components more thoroughly, the W–C–3Cu mixture should be milled for a longer duration. After 10 min of MM, large particles of tungsten were not detected in the W–C–3Cu mixture (Figure 1b). 

The cross-sectional images of the W–C–3Cu agglomerates show the areas of tungsten (light gray), copper (gray), and carbon black particles (black) (Figure 1c,d). With an increase in the milling duration, the distribution of the components in the agglomerates becomes more uniform. The particles of carbon black were distributed mainly in the copper matrix.

**Figure 2:**
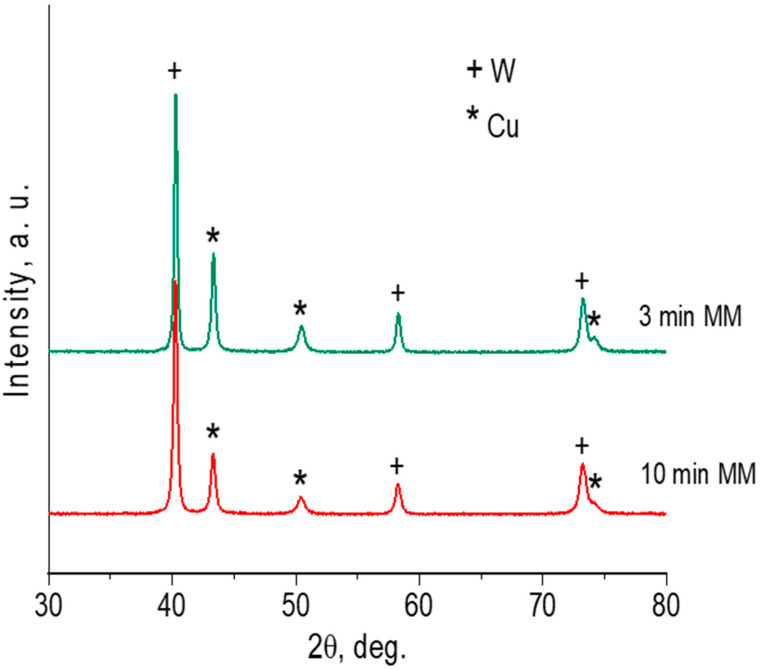
XRD patterns of W–C–3Cu powders milled for 3 min and 10 min.

The XRD patterns of the milled mixtures show the presence of metallic Cu and W (Figure 2). No reflections of carbon can be detected on the patterns because of its relatively low concentration in the mixture. The broadening of the W and Cu reflections after 10 min of MM indicates a decrease in the crystallite size and the lattice strain accumulation, which is demonstrated by the results of the crystalline structure refinement (Table 2). The lattice parameters of W and Cu were equal to those of pure metals, confirming the absence of the mutual dissolution of the components during milling. The crystallite sizes of Cu and W phases were smaller than 100 nm, which indicates the formation of nanostructured agglomerates. There were no reflections of possible reaction products (carbides) on the XRD patterns. However, the products can be present in the milled powders in small amounts not detectable by the XRD analysis.

**Figure 3:**
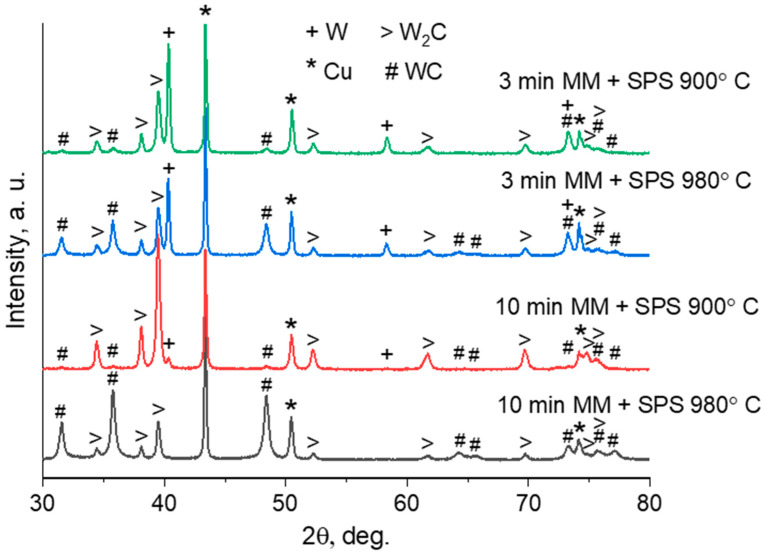
XRD patterns of the compacts consolidated by SPS from the mechanically milled W–C–3Cu powders.

The XRD patterns of the sintered materials show W, WC, W_2_C, and Cu reflections (Figure 3). The estimated concentrations of phases in the compacts are shown in Table 3. The reflections of W are absent only on the XRD pattern of the compact obtained by milling for 10 min and SPS at 980 °C. The composition of this compact is WC–W_2_C–Cu–C, which means a complete conversion of tungsten into the carbide phases. The presence of the W_2_C phase implies that unreacted carbon remained in the sample. In all other cases, the SPS of the mechanically milled W–C–3Cu mixtures does not result in a full conversion of W to the carbides. The key factor hindering the in situ solid-state synthesis of WC–Cu composites is the absence of the mutual solubility of both tungsten and carbon in the copper matrix. During SPS, in agglomerates obtained by MM, the formation of the WC and W_2_C carbides begins at the boundaries between the tungsten and carbon particles. Thus, after a longer MM, due to the formation of new contacts between the reactants, the synthesis of carbides occurs at a greater number of locations. The XRD patterns of the samples sintered at 900 and 980 °C from the mixture milled for 3 min show strong W reflections, indicating the presence of the starting reactants in the consolidated materials. The reflections of the WC phase become more intense when the sintering temperature is increased from 900 to 980 °C for both mixtures. This means that the formation of WC (Equations (2) and (3)) occurs faster at 980 °C.

Table 4 shows the lattice parameters, crystallite sizes, and lattice strain of the phases in the sintered compacts. It can be seen that, during SPS, nanostructured WC and W_2_C were synthesized in the W–C–3Cu mixtures. The crystallites of copper and tungsten in the compacts are larger than those of the carbides. The deviation of the lattice parameters of the WC and W_2_C carbides from the theoretical values indicates their non-stoichiometric composition. The W_2_C carbide has three different modifications: β-W_2_C having a hexagonal structure, β′-W_2_C having an orthorhombic structure, and β″-W_2_C having an rhombohedral structure. It is difficult to distinguish the XRD patterns of the β-W_2_C and β′-W_2_C phases. As noted in [22,23], the β′-W_2_C phase exists in samples annealed at temperatures below 1300 K. In the present work, we assumed that the synthesized W_2_C has an orthorhombic structure (β′-W_2_C).

**Figure 4:**
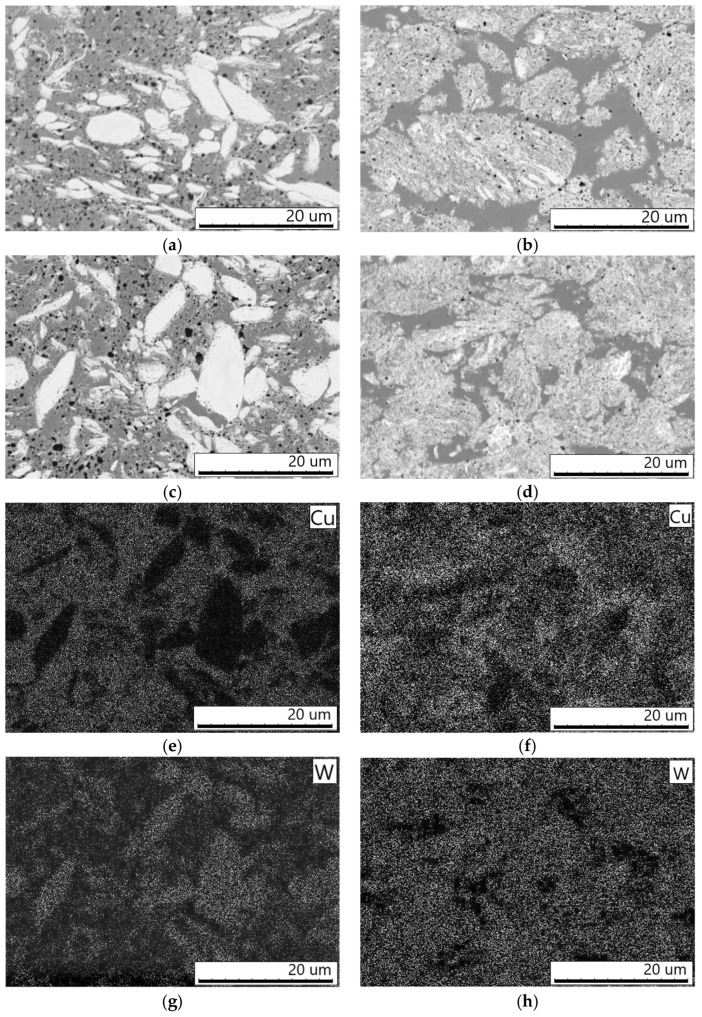
Microstructure of the compacts obtained by the SPS of the W–C–3Cu mixtures: (**a**) 3 min MM—900 °C, (**b**) 10 min MM—900 °C, (**c**) 3 min MM—980 °C, and (**d**) 10 min MM—980 °C. (**e**,**g**) Elemental maps of the area shown on (**b**) and (**f**,**h**) elemental maps of the area shown on (**d**).

Figure 4a–d show the microstructure of the compacts obtained by the SPS of the W–C–3Cu mixtures. The residual porosity of the compacts was less than 5%, according to the image analysis. In the structure of materials sintered from mixtures milled for 3 min (Figure 3a,c), light gray areas can be observed, which are W-rich according the elemental mapping (Figure 4e,g). As determined by the XRD, these compacts contained metallic tungsten. It appears that the light gray regions were mostly tungsten, kept from the powder state. In the Cu-rich areas (gray), submicron particles of carbon black were distributed (black). The tungsten carbides WC and W_2_C confirmed by XRD are contained in the compacts as fine particles located at the W/C interfaces. The carbide particles were not visible at the selected magnification. The SPS of the W–C–3Cu mixtures milled for 10 min led to formation of the materials with an interesting structure (Figure 4b,d). Fine-grained regions kept from the composite agglomerates formed by MM were visible. Between the composite regions, Cu-rich areas (gray) were located. The difference in the content of tungsten between the specific areas of the structure was confirmed by the elemental mapping (Figure 4f,h). The composite regions contain all phases, the reflections of which are present in the XRD patterns. There were no particles of carbon black in the Cu-rich areas, which are seen on the cross-sections of the corresponding particles of the milled mixture. The formation of Cu-rich regions is a result of local heating above the melting point of copper (1083 °C) and melting the material during SPS. The reason of local overheating is high-density electric currents at the inter-particle contacts. The copper melt filled the gaps between the agglomerates forming this unconventional structure. We previously studied this effect in detail for the case of reactive SPS of Ti–C–3Cu milled mixtures [11].

**Figure 5:**
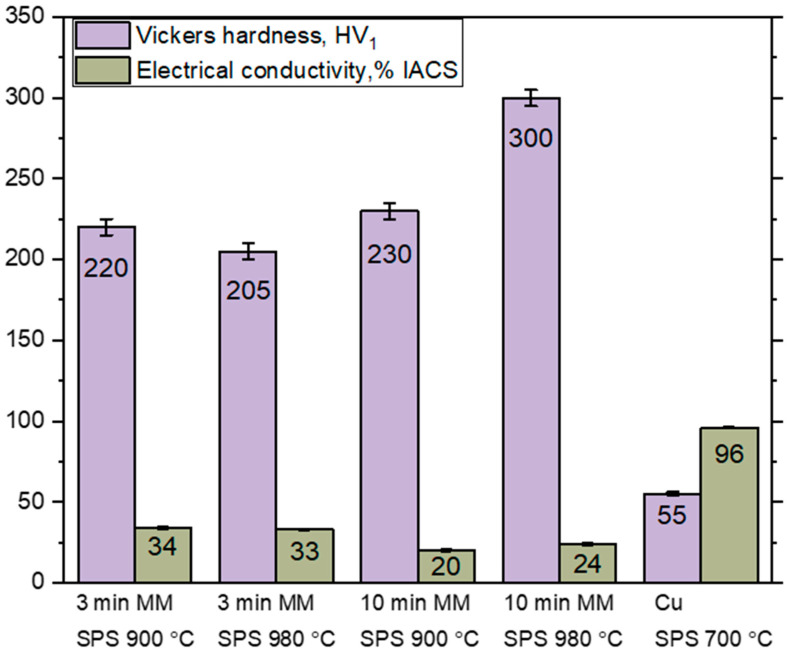
Electrical conductivity and hardness of the compacts obtained by the SPS of the mechanically milled W–C–3Cu powder mixtures and unmilled copper powder.

The electrical conductivity and hardness of a MMC depend on the concentration, size, and distribution of the reinforcing phase, impurity atoms in the matrix, crystallite sizes of the phases, and the porosity of the material. The hardness of the composite materials obtained in the present work was significantly higher than the hardness of sintered copper (Figure 5). The sample obtained by MM for 10 min and SPS at 980 °C demonstrated the highest hardness (300 HV_1_) in the studied series. In this compact, metallic tungsten was completely converted into the WC and W_2_C phases, which provided a high level of strengthening. The electrical conductivity of this WC–W_2_C–Cu sintered material is 24% IACS. In [12,24], materials with close compositions (53 wt.% WC–Cu and 40 wt.% WC–Cu) were manufactured. A nanocomposite of the 53 wt.% WC–Cu composition [24] exhibits a hardness of 426 HV and an electrical conductivity of 21% IACS. It was obtained by a complex four-step technology, including melting under pressure. The way proposed in the present work is simpler and allows reaching an excellent combination of properties. The spark plasma sintered ex situ 40 wt.% WC–Cu composite [12] showed a hardness of 226 HV and an electrical conductivity of ~40% IACS. In the approach used in the present work, the in situ synthesis of tungsten carbides in the mechanically milled mixtures is a way to improve the hardness of the material. A high level of electrical conductivity was ensured by the Cu-rich areas formed due to a local melting during SPS.

The compacts obtained by MM for 3 min and subsequent SPS exhibited an electrical conductivity of 33–34% IACS and a hardness of 205–220 HV_1_. These samples contained a high concentration of metallic W and were, therefore, softer and more conductive than the compacts sintered from the mixture milled for 10 min. Another factor contributing to a reduced hardness and an increased electrical conductivity was the large crystallite size of the copper matrix.

**Figure 6:**
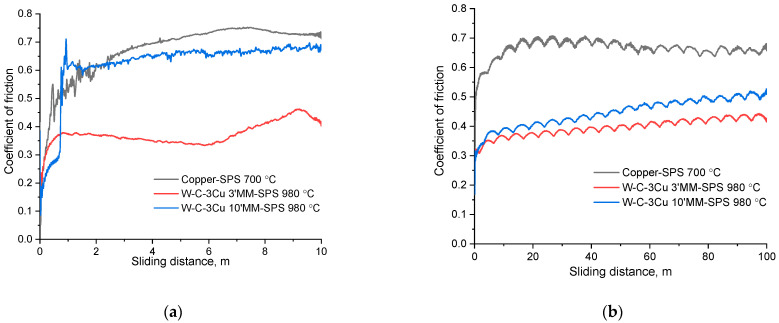
Coefficient of friction in the dry sliding wear tests: (**a**) in mode 1 using a steel ball and (**b**) in mode 2 using a WC-6Co ball.

**Figure 7:**
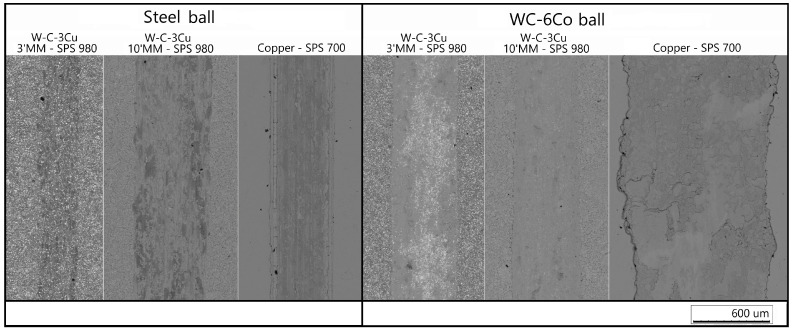
SEM images of the wear scars on the surfaces of the compacts after dry sliding wear.

**Figure 8:**
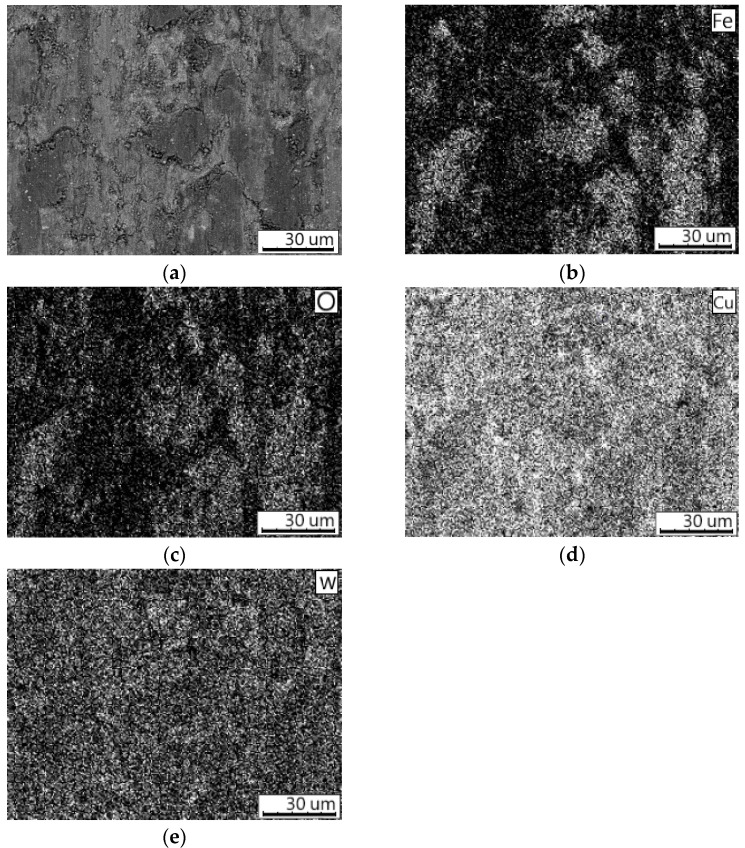
(**a**) The morphology of the worn surface of the compact obtained from the W–C–3Cu mixture milled for 10 min by SPS at 980 °C after a dry sliding wear test using the steel counter-body; (**b**–**e**) elemental maps of the area shown on (**a**).

**Figure 9:**
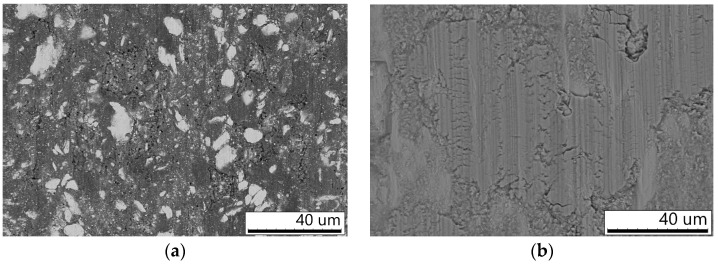

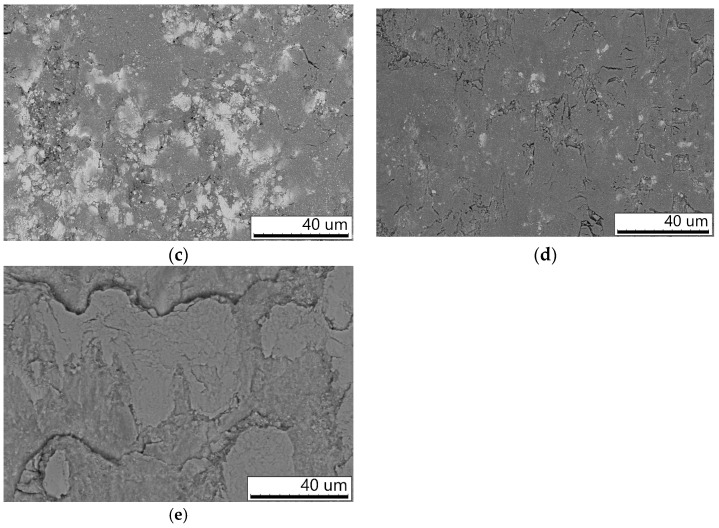
The morphology of the worn surface of the sintered compacts after dry sliding wear tests: (**a**) W–C–3Cu, 3 min MM, SPS 980 °C, steel ball; (**b**) copper, SPS 700 °C, steel ball; (**c**) W–C–3Cu, 3 min MM, SPS 980 °C, WC-6Co ball; (**d**) W–C–3Cu, 10 min MM, SPS 980 °C, WC-6Co ball; (**e**) and copper, SPS 700 °C, WC-6Co ball.

The wear behavior of the sintered compacts was evaluated with reciprocating sliding tests using two different counter-bodies: AISI 52100 steel and WC-6Co balls. Figure 6 shows the COFs values of the tested samples. It can be seen that the COF curves obtained using a steel ball (Figure 6a) were less stable compared with the curves obtained using a WC-6Co ball (Figure 6b). This behavior can be explained by the active transfer of the material of the steel balls to the surface of the examined samples. It is evident that the wear scars formed by the steel ball have a dark contrast in the SEM images (Figure 7). Figure 8a shows the worn surface of the compacts sintered from the mixture milled for 10 min. The elemental maps indicate that the distribution of Fe and O atoms on the sliding surfaces was the same. This can be explained by the formation of iron oxides on the surfaces. During testing, the energy dissipation caused a significant overheating of the contact zone and oxidation of the worn surface. The same behavior was observed for the compact consolidated from the mixture milled for 3 min. The worn surface of this sample is shown in Figure 9a. Despite its high hardness of AISI 52100 steel, the ball wears against the surface of the sample.

The COF values of the composites, irrespective of the counter-body material, were lower than those of sintered copper. Figure 9b shows strong stretches across the copper specimen after the test with the steel ball. On the worn surface of copper, an excessive plastically deformed metal was observed as a result of sliding by the WC-6Co ball (Figure 9e). Due to extremely high ductility and low hardness, the copper was worn off intensively. In the sintered composites, the tungsten carbide inclusions resisted the plowing effect of the counter-body, protected the material from destruction, and enhanced its wear resistance. In pairs with the steel ball, the W–WC–W_2_C–Cu–C compact (3 min MM—SPS 980 °C) showed the lowest COF value (0.37). The COF value of the sample obtained from the mixture milled for 10 min MM by SPS at 980 °C (WC–W_2_C–Cu–C composition) in a pair with steel was 0.62. This difference can be explained by the residual carbon black acting as a solid lubricant and decreasing COF. The concentration of residual carbon is higher in the composite obtained from the mixture milled for 3 min.

The mean COF values for the composite samples tested using a WC-6Co ball are 0.39 (3 min MM, SPS 980 °C) and 0.46 (10 min MM, SPS 980 °C). The COF values obtained in this work were about two times lower than those obtained for composites produced by the solid-state sintering of a mixture of Cu and WC powders [20]. The COF values recorded in the present work were on the level of those of the specially designed WC/Cu self-lubricating textured coatings [25]. In the 10 min MM—SPS 980 °C compact (WC–W_2_C–Cu–C composition), the hard composite regions enriched by WC and W_2_C phases and soft re-solidified copper areas were present. It is suggested that copper forms a film on the worn surface improving the wear resistance and acting as a solid lubricant. In the 3 min MM—SPS 980 °C compact (W–WC–W_2_C–Cu–C composition), a low COF value was achieved thanks to the simultaneous presence of residual carbon black, a soft copper matrix, and hard tungsten carbide particles. On the worn surfaces obtained after testing using a WC-6Co ball (Figure 9c,d), the plastically deformed copper layers were visible (dark gray), which indicates that soft copper covers the sliding surface. The specific wear rate values of the composite materials obtained in the present study (Table 5) were low and close to the values given in [25]. So, the metal–ceramic composite materials obtained in the present study possessed a high wear resistance. The volume losses after the tests with a steel ball in mode 1 were insignificant and difficult to measure.

Different wear mechanisms can act operate the dry sliding of MMCs, including adhesive wear, abrasive wear, oxidative wear, and delamination [26,27,28,29]. The adhesive wear involves the transfer of material from one surface to another by solid-state welding. For materials obtained in the present work, the adhesive wear mechanism can be operative due to a high concentration of copper. The abrasive wear results from the destruction of the material via friction by hard surfaces/particles. For the abrasive wear resistance of MMCs, the capability of the reinforcement pull-out is very important. The detached hard particles become new abrasive components and act as a third body, reducing the wear resistance. According to [30], composites reinforced by small ceramic particles exhibited a high abrasive wear resistance due to a high surface area of these particles and a developed particle/matrix interface. Other factors influencing the abrasive wear resistance of MMCs are the interfacial bond quality and the particle/matrix cohesion [30]. The approach of in situ synthesis used in the present work enables the formation of the submicron reinforcing WC and W_2_C particles and ensures the high purity of particle/matrix interfaces. It is suggested that, due to their high bonding strength, the particles remained embedded within the metallic matrix, improving the wear resistance of the composites. However, the carbide particles bonded together with soft copper can break off the surface during sliding. The abrasive wear can operate in the case of tests with a WC-6Co ball. Additionally, the oxidation of the worn surfaces was observed, indicating the action of the oxidative wear mechanism. On the one hand, oxidation leads to a growth of a protective tribo-oxide layer, reducing wear [31]. On the other hand, the delaminated hard oxide particles can participate in the surface destruction, which increases wear [31]. Summarizing the above, the main wear mechanisms of the sintered composites include adhesive, oxidative, and abrasive wear.

## 4. Conclusions

In this work, the reactive SPS of W, C, and Cu elemental powder mixtures prepared by MM was conducted. It was shown that the milling time is an important parameter for the subsequent synthesis of tungsten carbides in a copper matrix, as, with increase in milling time, the interfacial area between the W and C reactants increases. A complete conversion of tungsten into the carbide phases was achieved by MM for 10 min and subsequent SPS at a temperature of 980 °C. The microstructure of the synthesized WC–W_2_C–Cu composite is unconventional: Cu-rich areas are located between the composite regions inherited from the agglomerates formed during MM. This structure was formed due to the local melting of copper due to high-density electric currents at the inter-particle contacts. A WC–W_2_C–Cu composite shows an attractive combination of properties: a hardness of 300 HV, an electrical conductivity of 24% IACS, a residual porosity less than 5%, a coefficient of friction in a pair with a WC-6Co ball of 0.46, and a specific wear rate of the material of 0.52 × 10^−5^ mm^3^ N^−1^ m^−1^.

## Figures and Tables

**Table 1:** Parameters of the dry sliding wear tests conducted under ball-on-flat reciprocating sliding conditions.

Parameters	Mode 1	Mode 2
Counter-body material	AISI 52100 steel	WC-6Co
Counter-body (ball) diameter, mm	6.35	6.35
Sliding frequency, Hz	1	5
Stroke length, mm	5	5
Applied normal load, N	5	25
Total sliding distance, m	10	100
Number of cycles	1000	2000

**Table 2:** Lattice parameters, crystallite size, and lattice strain of the W–C–3Cu powders mechanically milled for 3 min and 10 min. The lattice parameter of pure copper is *a* = 3.615 Å (crystallography open database (COD) number of 4105681). The lattice parameter of pure tungsten is *a* = 3.165 Å (COD number of 9008558). MM—mechanical milling.

Powder	Cu Lattice Parameter (*a)*, Å	Cu Crystallite size, nm	Cu Lattice Strain,%	W Lattice Parameter (*a)*, Å	W Crystallite Size, nm	W Lattice Strain,%
W–C–3Cu, 3 min MM	3.615 ± 0.003	36 ± 1	0.25 ± 0.03	3.165 ± 0.002	64 ± 1	0.41 ± 0.02
W–C–3Cu, 10 min MM	3.615 ± 0.002	27 ± 1	0.55 ± 0.03	3.165 ± 0.001	42 ± 1	0.44 ± 0.01

**Table 3:** Concentrations of phases in the compacts obtained by the SPS of the mechanically milled W–C–3Cu powders determined via the Rietveld refinement.

Processing Conditions	Concentration, wt.%				
	Cu	W	W_2_C	WC	C *
3 min MM, SPS 900 °C	57.2	16.4	21.3	3.4	1.7
3 min MM, SPS 980 °C	51.9	11.5	14.4	21.0	1.2
10 min MM, SPS 900 °C	51.0	1.1	44.7	1.7	1.5
10 min MM, SPS 980 °C	52.2	-	10.6	36.9	0.3

* Calculated using the concentrations of the WC and W_2_C phases determined via the Rietveld analysis.

**Table 4:** Lattice parameters, crystallite sizes, and lattice strain of the compacts obtained by the SPS of the milled W–C–3Cu powders. The lattice parameter of pure copper is *a* = 3.615 Å (COD number of 4105681). The lattice parameter of pure tungsten are *a* = 3.165 Å (COD number of 9008558). The lattice parameters of WC are *a* = 2.906 Å and *c* = 2.838 Å (COD number of 1501516). The lattice parameters of W_2_C (*Pbcn*) are *a* = 4.721 Å, *b* = 6.03 Å, and *c* = 5.18 Å (COD number of 108194).

Processing Conditions	Cu Lattice Parameter (*a*), Å			Cu Crystallite Size, nm	Cu Lattice Strain,%	W Lattice Parameter (*a*), Å		W Crystallite Size, nm	W Lattice Strain,%
W–C–3Cu, 3 min MM, 900 °C	3.616 ± 0.003			105 ± 2	0.12 ± 0.01	3.166 ± 0.003		87 ± 1	0.31 ± 0.01
W–C–3Cu, 3 min MM, 980 °C	3.617 ± 0.002			110 ± 2	0.10 ± 0.01	3.167 ± 0.002		83 ± 1	0.25 ± 0.02
W–C–3Cu, 10 min MM, 900 °C	3.618 ± 0.003			77 ± 1	0.14 ± 0.01	3.166 ± 0.003		59 ± 2	0.39 ± 0.01
W–C–3Cu, 10 min MM, 980 °C	3.619 ± 0.003			93 ± 3	0.19 ± 0.01	-		-	-
**Processing** **Conditions**	**W_2_C Lattice Parameter (*a*), Å**	**W_2_C Lattice Parameter (*b*), Å**	**W_2_C Lattice Parameter (*c*), Å**	**W_2_C** **Crystallite Size, nm**	**W_2_C** **Lattice Strain,%**	**WC Lattice Parameter (*a*), Å**	**WC Lattice Parameter (*c*), Å**	**WC** **Crystallite Size, nm**	**WC** **Lattice Strain,%**
W–C–3Cu, 3 min MM, 900 °C	4.731 ± 0.001	6.043 ± 0.001	5.197 ± 0.001	49 ± 1	0.3 ± 0.03	2.900 ± 0.001	2.843 ± 0.001	22 ± 2	0.35 ± 0.02
W–C–3Cu, 3 min MM, 980 °C	4.731 ± 0.001	6.043 ± 0.001	5.195 ± 0.001	56 ± 2	0.22 ± 0.04	2.903 ± 0.001	2.843 ± 0.001	24 ± 1	0.23 ± 0.06
W–C–3Cu, 10 min MM, 900 °C	4.733 ± 0.001	6.045 ± 0.001	5.204 ± 0.001	51 ± 1	0.29 ± 0.02	2.900 ± 0.001	2.845 ± 0.001	24 ± 1	0.45 ± 0.1
W–C–3Cu, 10 min MM, 980 °C	4.732 ± 0.001	6.043 ± 0.001	5.202 ± 0.001	48 ± 2	0.13 ± 0.08	2.903 ± 0.001	2.844 ± 0.001	24 ± 1	0.23 ± 0.04

**Table 5:** Volume loss and specific wear rate of the compacts in the dry sliding wear test using a WC-6Co counter-body.

Composition and Processing Conditions	Volume Loss, mm^3^	Specific Wear Rate ∙ 10^−5^, mm^3^∙N^−1^∙m^−1^
W–C–3Cu, 3 min MM, 980 °C	0.013 ± 0.007	0.52 ± 0.28
W–C–3Cu, 10 min MM, 980 °C	0.013 ± 0.003	0.52 ± 0.12
Copper, 700 °C	0.068 ± 0.019	2.72 ± 0.76

## Data Availability

The data presented in this study are available upon request from the corresponding authors.
